# Activation of conventional and plasmacytoid dendritic cells and enhancement of anti-viral immune responses by *Lactobacillus delbrueckii* subsp. *bulgaricus* OLL1073R-1 and *Streptococcus thermophilus* OLS3059

**DOI:** 10.1371/journal.pone.0359545

**Published:** 2026-09-29

**Authors:** Hiroki Kono, Toshihiro Sashihara

**Affiliations:** Wellness Science Labs, Meiji Holdings Co., Ltd., Hachioji, Tokyo, Japan; Kansai Medical University: Kansai Ika Daigaku, Institute of Biomedical Science, JAPAN

## Abstract

Dendritic cells are classified into conventional dendritic cells (cDCs) and plasmacytoid dendritic cells (pDCs), both of which play critical roles in anti-viral immune responses. Previous studies have reported that dairy consumption of yogurt fermented with *Lactobacillus delbrueckii* subsp. *bulgaricus* OLL1073R-1 and *Streptococcus thermophilus* OLS3059 had the effect of inhibiting viral infections. Recent studies have shown that the activation of both cDCs and pDCs and the decrease of respiratory symptoms were induced by the consumption of this yogurt. However, the components responsible for these effects and the underlying mechanisms remain unclear. Since several reports have shown that cDCs and pDCs were activated by lactic acid bacteria, we hypothesized *L*. *bulgaricus* OLL1073R-1 and *S. thermophilus* OLS3059 induced anti-viral immune responses by activating cDCs and pDCs. Here we demonstrated that both *L*. *bulgaricus* OLL1073R-1 and *S. thermophilus* OLS3059 activate cDCs and pDCs in human peripheral blood mononuclear cells. We also showed that both cDCs and pDCs were necessary for type I interferon production, whereas cDCs, but not pDCs, were essential for enhancing effector memory CD4(+) T cell responses to viral antigens when stimulated by *L*. *bulgaricus* OLL1073R-1. These findings indicate that anti-viral immune responses induced by consumption of this yogurt are mediated by both *L*. *bulgaricus* OLL1073R-1 and *S. thermophilus* OLS3059 through activation of cDCs and pDCs.

## Introduction

Dendritic cells (DCs) are the central regulators of the immune system, bridging innate and adaptive immunity by recognizing pathogens through various receptors and inducing antigen-specific responses and immune memory [1]. Human DCs differentiate from CD34(+) hematopoietic progenitor cells into two major subsets: conventional dendritic cells (cDCs) and plasmacytoid dendritic cells (pDCs), which migrate into blood and peripheral tissues [2,3]. cDCs are predominant DC subset and are responsible for activating T cells by antigen presentation or cytokine production [3,4]. In contrast, pDCs are characterized by producing large amounts of type I interferons (IFNs) in response to pathogens [3,5].

DCs have been reported to play an important role in the control of infectious diseases as well as in the pathogenesis of various immune diseases including autoimmune diseases, allergy, cancer [6,7]. cDCs recognize viruses, bacteria and fungus through a variety of pattern recognition receptors such as Toll-like receptors (TLRs) [8]. pDCs highly express TLR7 and TLR9 which are known as intracellular receptors and recognize viral single-stranded RNA and CpG rich DNA [8]. Several reports have shown that experimental depletion of cDCs leads to increase pathogen burden and reduce survival rate during infection of viruses or bacteria [9–12]. Similarly, depletion of pDCs has been shown to impair antigen-specific antibody production during viral infections [10]. In humans, both cDCs and pDCs have been also reported to be related to induction of immune responses and clearance of pathogens during infections [13,14]. Collectively, these findings suggest that the activation of cDCs and pDCs plays a critical role in suppressing infection of various viruses or bacteria.

Several reports have shown that the consumption of probiotic drink containing lactic acid bacteria decrease the frequency of catching respiratory infectious diseases [15–17]. Our studies have shown that immunomodulatory effects such as activation of natural killer cells and improvement of respiratory symptoms were induced by the consumption of the yogurt fermented with *Lactobacillus delbrueckii* subsp. *bulgaricus* OLL1073R-1 (hereafter referred as OLL1073R-1) and *Streptococcus thermophilus* OLS3059 (hereafter referred as OLS3059) [18–22]. We have also reported by using influenza infection mouse model that intake of this yogurt reduced viral load and improved survival rate [23]. We have shown in human study that influenza-reactive salivary immunoglobulin A (IgA) and serum IgG levels after influenza vaccination were increased by the consumption of this yogurt [24]. These findings collectively indicate that suppression of viral infection is mediated by the consumption of yogurt fermented with OLL1073R-1 and OLS3059.

It has been reported that cDCs and pDCs are activated by several kinds of lactic acid bacteria *in vitro* experiments [25–28]. Our recent human studies have shown that both activation of cDCs and pDCs and improvement of respiratory symptoms are induced by daily consumption of the yogurt [20,21]. Based on these observations, we hypothesized that OLL1073R-1 and OLS3059 contained in the yogurt activated cDCs and pDCs and thereby enhanced anti-viral immune responses. In the present study, we used human peripheral blood mononuclear cells (PBMCs) to clarify whether cDCs and pDCs were activated by OLL1073R-1 and OLS3059 and determine how such activation contributes to anti-viral immune responses. Through this approach, we aimed to understand whether and how lactic acid bacteria induce anti-viral immune responses by acting on cDCs and pDCs.

## Materials and methods

### Preparation of lactic acid bacterial cells

*L*. *bulgaricus* OLL1073R-1, JCM 1002^T^, MH250033, MH250034 were cultured by using Difco™ Lactobacilli De Man-Rogosa-Sharpe (MRS) Broth (BD, New Jersey, USA) and *S. thermophilus* OLS3059, JCM 17834^T^, MH260001, MH260002 were cultured by Difco™ M17 Broth (BD) at 37°C, anaerobic, state condition for 18 h after preculture of the cryopreserved stocks. Bacteria were washed twice by phosphate-buffered saline (PBS) and were heat-treated at 75°C for 1 h and were preserved at −80°C until used in following experiments. The number of bacteria was calculated by counting colonies on Difco™ Lactobacilli MRS Agar (BD) using bacteria suspension before heat-treated. Strains OLL1073R-1, MH250033, MH250034, OLS3059, MH260001 and MH260002 were obtained from Meiji Co., Ltd. (Tokyo, Japan) Strain JCM 1002^T^ and JCM 17834^T^ was purchased from Japan Collection of Microorganisms (Tsukuba, Japan).

### Stimulation of PBMCs with lactic acid bacteria

Human PBMCs (Precision for Medicine, Bethesda, USA) were seeded at a concentration of 5 × 10^5^ cells/well and cultured in 200 μL medium at 37°C, 5% CO_2_ with or without lactic acid bacteria at a concentration of 1 × 10^6^ bacterial cells/mL for 24 h. Roswell park memorial institute (RPMI) 1640 Medium (Thermo Fisher Scientific, Massachusetts, USA) containing 10% fetal bovine serum (FBS) was used for incubation. Cells were stained by anti-human lineage cocktail 1 fluorescein isothiocyanate (FITC) (BD), anti-human leukocyte antigen (HLA) -DR allophycocyanin (APC) (BD), anti-CD11c brilliant violet (BV) 421 (BD), anti-CD123 BV510 (BD), anti-CD304 phycoerythrin (PE) (BD), anti-CD86 PE-Cyanine (Cy) ™7 (BD), anti-CD40 peridinin chlorophyll protein (PerCP) -Cy™5.5 (Biolegend, San Diego, USA), Fixable Viability Dye eFluor™ 780 (Thermo Fisher Scientific) after blocking with Human BD Fc Block™ (BD) and were fixed and permeated by BD Cytofix/Cytoperm™ Fixation/Permeabilization Kit (BD) and were analyzed CD86, HLA-DR, CD40 expression of cDCs or pDCs fraction by BD FACS Lyric™ (BD).

When performing intracellular staining of IL-12, BD GolgiStop™ Protein Transport Inhibitor (BD) was added 2 h after cultivation of PBMCs with lactic acid bacteria when intracellular staining of interleukin (IL) −12 was performed. Cells were stained by anti-human lineage cocktail 1 FITC (BD), anti-HLA-DR APC (BD), anti-CD11c BV421 (BD), anti-CD123 BV510 (BD), anti-CD304 PE (BD), Fixable Viability Dye eFluor™ 780 (Thermo Fisher Scientific) after blocking and were fixed and permeated and were stained by anti-IL-12 PE (BD) and were analyzed IL-12 expression of cDCs fraction by BD FACS Lyric™ (BD).

### Differentiation of DCs and sorting of cDCs and pDCs fraction

The differentiation from CD34(+) hematopoietic progenitor cells (HPCs) into cDCs and pDCs was performed according to previous study [29]. Human cord blood CD34(+) cells (Stemcell Technologies, Vancouver, Canada) were cultured at 37°C, 5% CO_2_ for 3 weeks by using X-VIVO15 medium (Lonza, Basel, Switzerland) with 10% FBS, 1% sodium pyruvate and 1% non-essential amino acids containing 20 ng/mL IL-3 (Thermo Fisher Scientific), 50 ng/mL thrombopoietin (TPO; Thermo Fisher Scientific) and 100 ng/mL Fms-like tyrosine kinase 3 ligand (Flt3-L; Thermo Fisher Scientific). Cells were stained by anti-human lineage cocktail 1 FITC (BD), anti-HLA-DR APC (BD), anti-CD11c BV421 (BD), anti-CD123 BV510 (BD), anti-CD304 PE (BD) after blocking and sorted cDCs and pDCs fractions by BD FACS Melody™ (BD). Sorted cDCs and pDCs fractions were defined as each “isolated cDCs” and “isolated pDCs”.

### Stimulation of isolated cDCs and pDCs with OLL1073R-1

Isolated cDCs and pDCs were each seeded at a concentration of 2 × 10^4^ cells/well and 5 × 10^4^ cells/well, respectively, and cultured in 200 μL medium at 37°C, 5% CO_2_ with or without OLL1073R-1 (1 × 10^6^ bacterial cells/mL) for 6 h or 24 h. RNA samples from cells incubated for 6 h were extracted by Maxwell® RSC simplyRNA Cells Kit (Promega, Madison, USA) and were used for mRNA-seq and qPCR. Cells incubated for 24 h were stained by anti-CD86 PE-Cy™7 (BD), anti-CD40 PerCP-Cy™5.5 (Biolegend), Fixable Viability Dye eFluor™ 780 (Thermo Fisher Scientific) after blocking and were fixed and permeated by BD Cytofix/Cytoperm™ Fixation/Permeabilization Kit (BD) and were analyzed CD86, HLA-DR, CD40 expression of live cells by BD FACS Lyric™ (BD). Supernatants of cells incubated for 24 h were used for quantification of IL-12, IFN-α2a and IFN-β levels.

### mRNA-seq of isolated cDCs and pDCs stimulated with OLL1073R-1

mRNA-seq library was prepared from RNA samples of isolated cDCs and pDCs by Illumina® Stranded mRNA Prep, Ligation kit (Illumina, San Diego, USA) and sequenced by Illumina Next-Seq 1000/2000 (Illumina), following conversion into FASTQ files by Dragen BCL Convert (Illumina). Gene annotation was performed using hg38 reference genome and read count data was transformed into transcript per million by Dragen RNA (Illumina). Comparison of gene expressions between OLL1073R-1-stimulated group and control group were performed by Dragen Differential Expression (Illumina). Differentially expressed genes (DEGs) were defined as followed: log_2_ fold change > |0.5| and p < 0.05. Enrichment analysis was performed in “regulation of cellular processes” pathway of Metacore (Clarivate, Philadelphia, USA) using genes upregulated in OLL1073R-1-stimulated group in DEGs.

### Stimulation of cDCs and pDCs-depleting PBMCs with OLL1073R-1

PBMCs were stained by anti-CD11c BV421 (BD) or anti-CD304 PE (BD) after blocking and sorted CD11c(-) fraction as “cDCs-depleted PBMCs” and CD304(-) fraction as “pDCs-depleted PBMCs” by BD FACS Melody™ (BD). PBMCs, cDCs-depleted PBMCs and pDCs-depleted PBMCs were each seeded at a concentration of 5 × 10^5^ cells/well and cultured in 200 μL medium at 37°C, 5% CO_2_ with or without OLL1073R-1 (1 × 10^6^ bacterial cells/mL), Influenza A (H1N1) virus-2 antigen (HyTest, Turku, Finland, 0.2 μg/mL). Supernatants of cells incubated without influenza antigen for 24 h were used for quantification of IFN-α2a and IFN-β. Supernatants of cells incubated with influenza antigen were collected for quantification of IL-12 at the point of 20 h after initiation and cells were incubated for 4 h longer with BD GolgiStop™ Protein Transport Inhibitor (BD). Cells were stained by anti-CD3 BV605 (Biolegend), anti-CD4 BV480 (BD), anti-CD8 PE-Cy™7 (BD), anti-CD45RA APC (BD), anti-CCR7 PE (BD), Fixable Viability Dye eFluor™ 780 (Thermo Fisher Scientific) after blocking and were fixed and permeated and were stained by anti-IFN-γ PE (BD) and were analyzed IFN-γ expression of effector-memory CD4(+) T cells (CD4(+) T_EM_) fraction by BD FACS Lyric™ (BD).

### Measurement of cytokine levels

IL-12 levels in the culture supernatants were quantified using an IL-12 by AuthentiKine™ IL-12 p40 ELISA Kit (Proteintech, Rosemont, USA). IFN-α2a and IFN-β levels were quantified by S-PLEX (Meso Scale Discocvery, Rockville, USA). Each experiment was performed according to manufacturers’ protocols. The minimum detectable concentrations were 15.6 pg/mL for IL-12, 4.9 fg/mL for IFN-α2a and 17.0 fg/mL for IFN-β.

### Detecting phagocytosis of OLL1073R-1 by cDCs and pDCs in PBMCs

PBMCs were seeded at a concentration of 5 × 10^5^ cells/well and cultured in 200 μL medium at 37°C, 5% CO_2_ with or without OLL1073R-1 (1 × 10^6^ bacterial cells/mL) labeled by pHrodo™ red AM Intracellular pH indicator (Thermo Fisher Scientific) for 24 h. Cells were stained by anti-human lineage cocktail 1 FITC (BD), anti-HLA-DR APC (BD), anti-CD11c BV421 (BD), anti-CD123 BV510 (BD), anti-CD304 PE (BD) after blocking and analyzed by Spectral Cell Analyzer ID7000 (Sony, Tokyo, Japan).

### Statistical analysis

All statistical analyses were performed by BellCurve for Excel ver. 3.20. Test of normality was conducted by Shapiro-Wilk test. Statistical comparisons were performed by Student’s t-test or Dunnett’s test for normally distributed data and Mann-Whitney U test for non-normally distributed data. All statistical tests were two-sided, and *p* < 0.05 was considered statistically significant. Each plot represents PBMCs or isolated DCs derived from one individual donor.

## Result

### OLL1073R-1 and OLS3059 activate cDCs and pDCs in PBMCs

We first investigated whether OLL1073R-1 and OLS3059 activated cDCs and pDCs in PBMCs. We selected CD86, CD40 and HLA-DR as activation markers of DCs, which were widely known as antigen presentation-related molecules and reported to be associated with DC activation [30,31]. PBMCs were stimulated with OLL1073R-1 or OLS3059 for 24 h, and expressions of activation markers in cDCs and pDCs were analyzed by flow cytometry. Gating strategies of cDCs and pDCs fractions in PBMCs were shown in S1 Fig. The results showed that CD86, CD40 and HLA-DR expression was not increased in cDCs but significantly increased in pDCs by stimulation with both OLL1073R-1 and OLS3059 (Fig 1A, B). The ratio of pDCs expressing these activation markers was also significantly increased upon stimulation with OLL1073R-1 or OLS3059 (S2 Fig).

**Fig 1:**
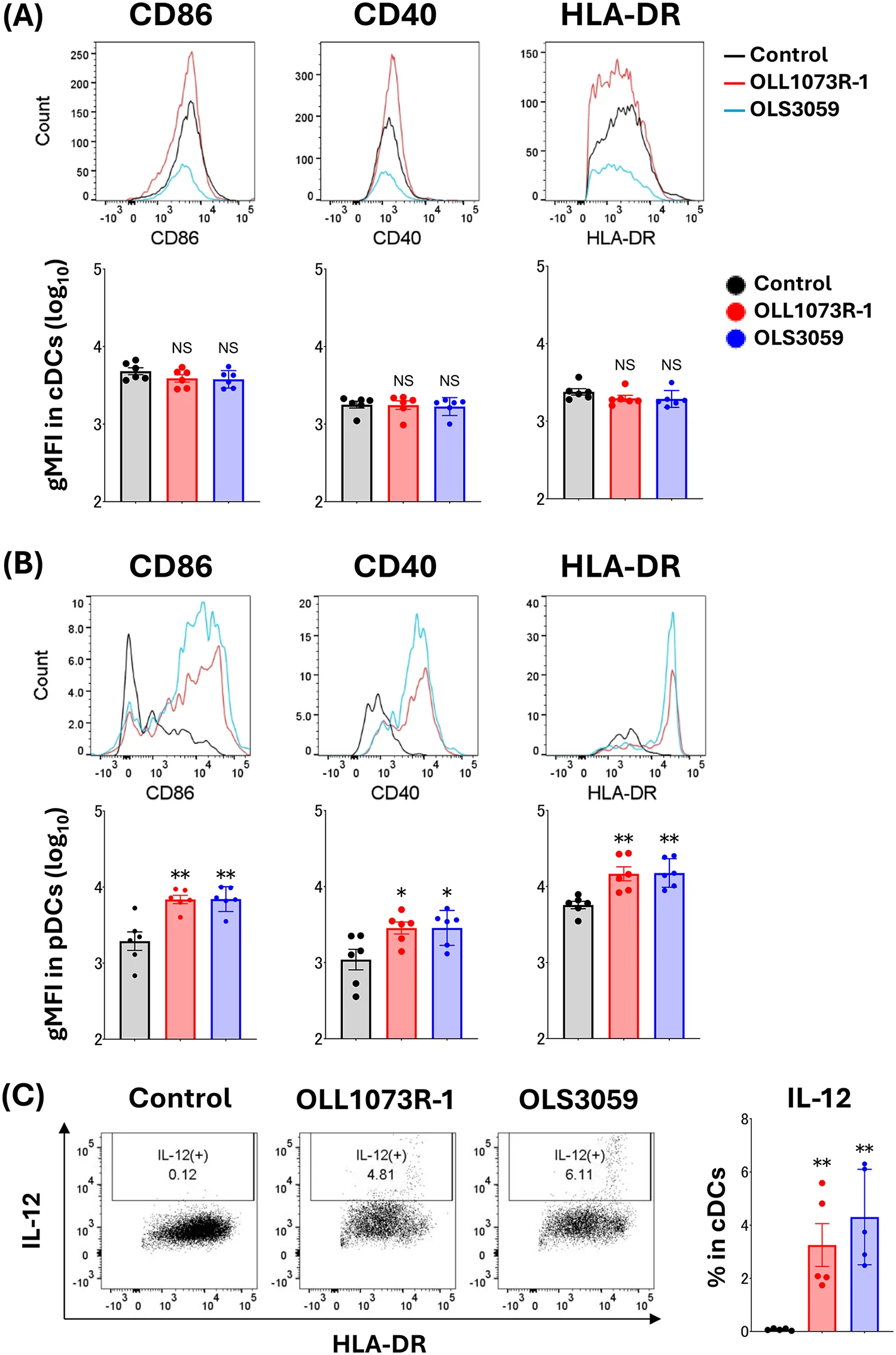
Activation of cDCs and pDCs in PBMCs by OLL1073R-1 and OLS3059. PBMCs are cultured with or without *L*. *bulgaricus* OLL1073R-1 or *S. thermophilus* OLS3059 for 24 h. The expression of CD86, CD40, and HLA-DR in (A) cDCs and (B) pDCs was analyzed by flow cytometry. Representative histograms (black, control; red, OLL1073R-1; blue, OLS3059) and the corresponding geometric mean fluorescent intensity (gMFI) are shown (n = 6). (C) Representative plots of IL-12 expression in cDCs were shown, and the ratio of IL-12 expressing cells in cDCs were analyzed (n = 5). *: *p* < 0.05, **: *p* < 0.01, NS: not significant by Dunnett’s test.

Previous reports have shown that the expression of antigen presentation-related molecules in cDCs was suppressed through interaction with regulatory T cells present in PBMC cultures [32]. Thus, we next assessed IL-12 expression as an alternative marker of cDC activation. Intracellular cytokine staining revealed that the ratio of IL-12 expressing cDCs was significantly increased by OLL1073R-1 or OLS3059 stimulation (Fig 1C). Collectively, these results demonstrated that OLL1073R-1 and OLS3059 activate both cDCs and pDCs in PBMCs.

### OLL1073R-1 directly activates cDCs and pDCs

Hereafter we focused on OLL1073R-1 and investigated whether the activation of these cells in PBMCs was due to direct effects on these cells or indirect effects mediated through other immune cells. cDCs and pDCs were differentiated from CD34(+) HPCs and subsequently sorted as shown in S3 Fig. The isolated cDCs and pDCs were stimulated with OLL1073R-1 for 24 h. The results showed that expression of CD86, CD40 and HLA-DR was significantly increased in both cDCs and pDCs, whereas HLA-DR expression showed only a trend toward an increase in cDCs following OLL1073R-1 stimulation (*p* < 0.1) (Fig 2A, B). The ratio of both cDCs and pDCs expressing these activation markers was also significantly elevated by stimulation with OLL1073R-1 (S4 Fig). These findings indicate that OLL1073R-1 directly activated cDCs and pDCs, independent of other immune cells.

**Fig 2:**
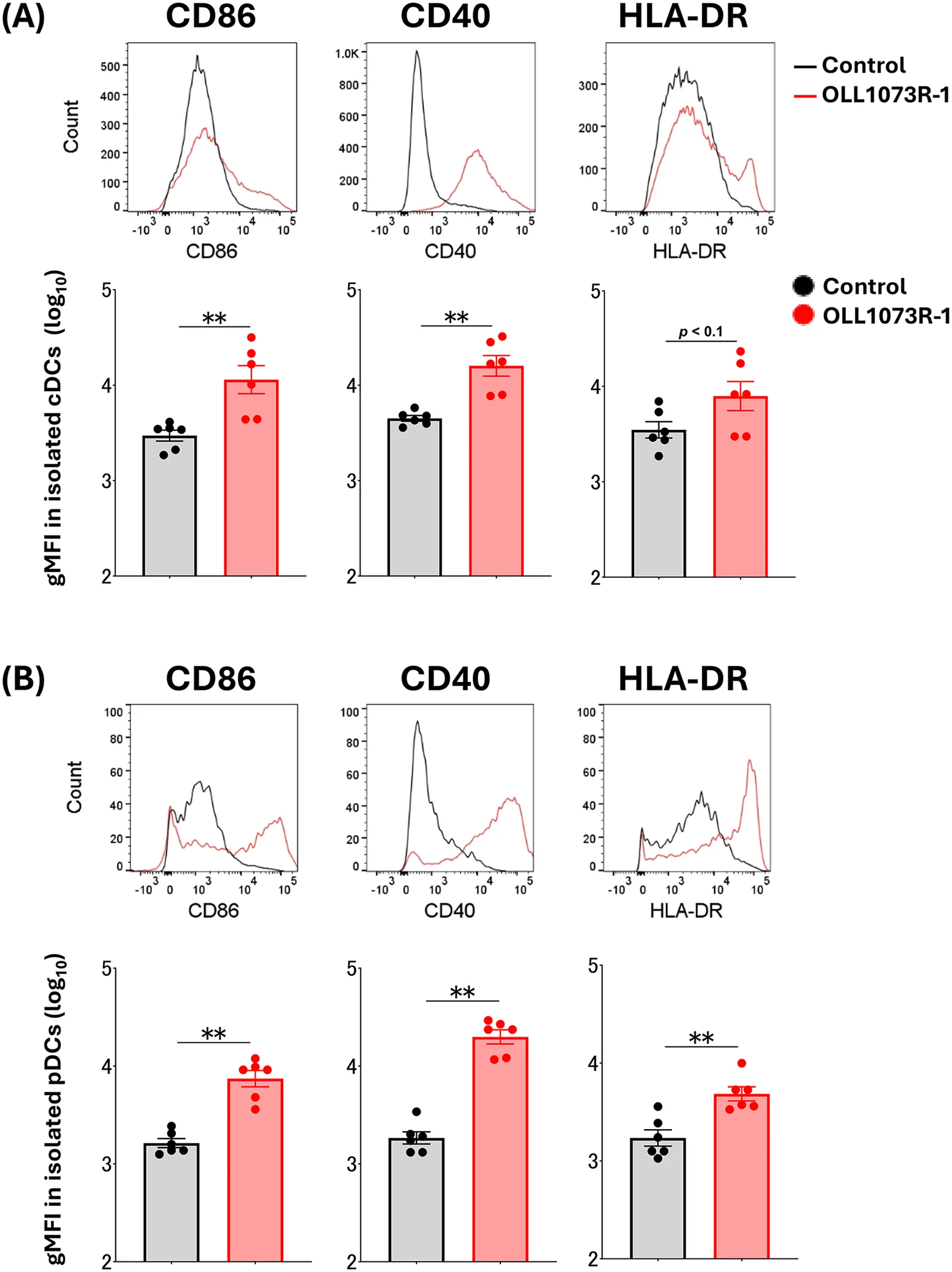
Activation of isolated cDCs and pDCs by OLL1073R-1. Isolated cDCs and pDCs are cultured with or without *L*. *bulgaricus* OLL1073R-1 for 24 h. The expression of CD86, CD40, and HLA-DR in (A) cDCs and (B) pDCs was analyzed by flow cytometry. Representative histograms (black, control; red, OLL1073R-1) and the corresponding geometric mean fluorescent intensity (gMFI) in live cells were shown (n = 6). **: *p* < 0.01 by Student’s t-test.

### RNA-seq analysis reveals immune pathways enhanced by OLL1073R-1 in cDCs and pDCs

It remained unclear whether activation of cDCs and pDCs by OLL1073R-1 contributes to anti-viral immune responses. In order to clarify this, we performed mRNA-seq of isolated cDCs and pDCs stimulated with OLL1073R-1. We used RNA samples of cDCs and pDCs stimulated by OLL1073R-1 for 6 h, as gene expression changes need to be captured at an earlier time point than phenotypic outcomes. Differential expression analysis identified 411 upregulated genes and 261 downregulated genes in cDCs, and 387 upregulated genes and 192 downregulated genes in pDCs (Fig 3A, B).

**Fig 3:**
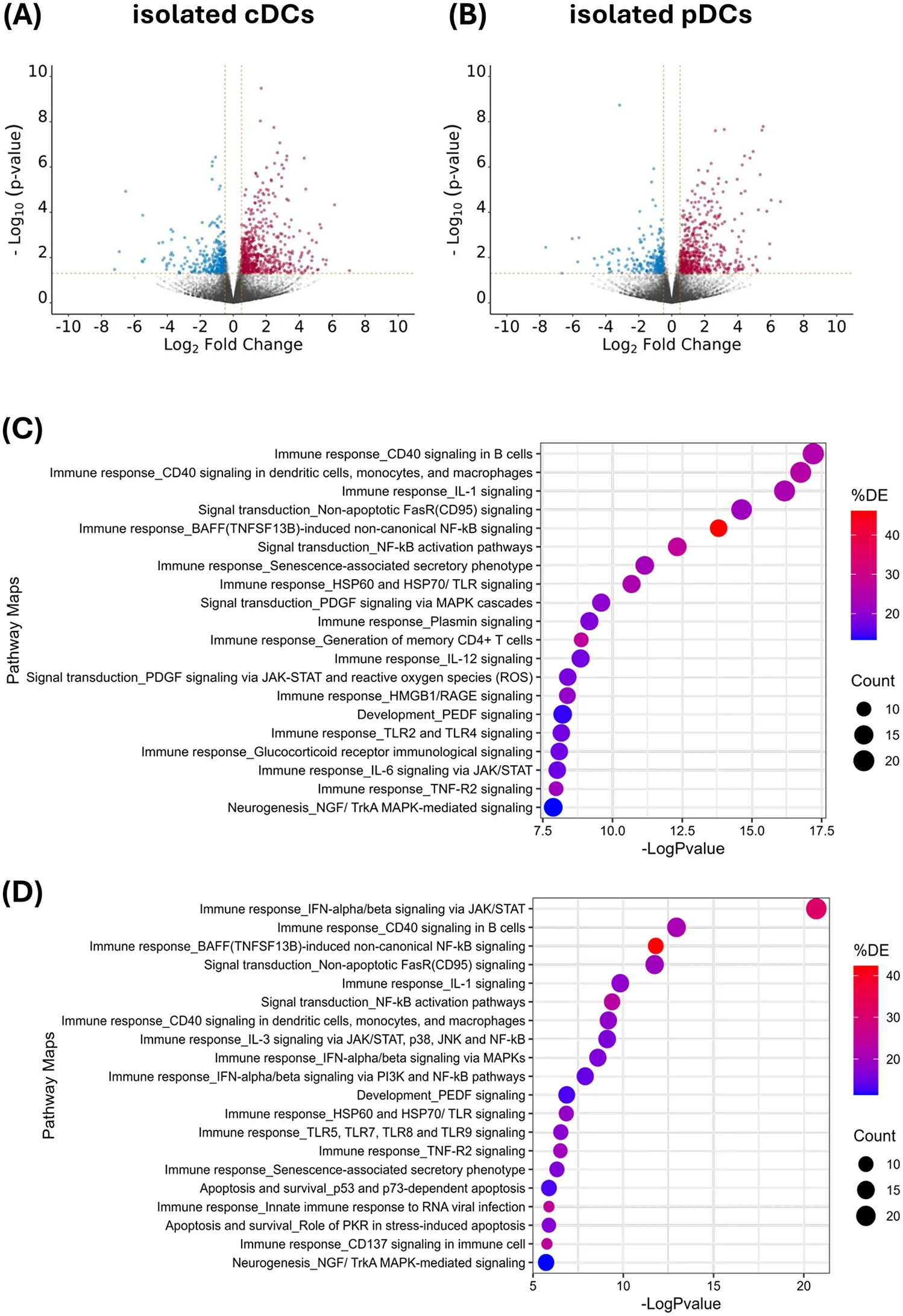
mRNA-seq of isolated cDCs and pDCs upon stimulation with OLL1073R-1. Isolated cDCs and pDCs are cultured with or without *L*. *bulgaricus* OLL1073R-1 for 6 h for mRNA-seq (n = 4). Volcano plots expressing differentially expressed genes (DEGs) in (A) cDCs and (B) pDCs were shown. Blue and red dots represent genes that were downregulated or upregulated (DEGs, |log_2_FC| > 0.5, *p* < 0.05), respectively, following stimulation with OLL1073R-1. Upper 20 enriched pathways of upregulated DEGs in (C) cDCs and (D) pDCs were shown. Count indicates the number of DEGs in each pathway. Percent DE indicates the percentage of DEGs in the total object in each pathway.

We performed enrichment analysis with genes upregulated with the stimulation of OLL1073R-1 and upper 20 pathways in each DC subsets were shown in Fig 3C, D. Pathways associated with CD40 signaling were ranked higher in both cDCs and pDCs, which indicated that activation of cDCs and pDCs were also confirmed by RNA-seq data. In addition, IL-1 and non-apoptotic CD95 signaling pathways, which have been reported to enhance CD40 expression, were also highly ranked [33,34]. Toll-like receptor (TLR) signaling pathways and downstream NF-κB signaling pathways were enriched in both DC subsets, suggesting that recognition of OLL1073R-1 by TLRs might be related to the activation of cDCs and pDCs.

Some pathways were characteristically enhanced either in cDCs or pDCs. Pathways related to CD4(+) T cell activation and IL-12 production were selectively enhanced in cDCs (Fig 3C). Since it is widely known that IL-12 contributes to activation of CD4(+) T cells [35], it is suggested that cDCs activated by OLL1073R-1 promote CD4(+) T cell responses through IL-12 production. In contrast, pathways related to IFN-α/β signaling were selectively enhanced in pDCs (Fig 3D). In the context of viral infection, type Ι IFNs are critical for anti-viral responses at early phase of infection, whereas CD4(+) T cells contribute to antigen specific responses and immunological memory at later phase of infection [36]. These findings suggest complementary role for cDCs and pDCs in anti-viral immune responses mediated by OLL1073R-1.

### cDCs and pDCs differently contribute to anti-viral immune responses induced by OLL1073R-1

We next examined whether cDCs and pDCs activated by OLL1073R-1 contribute to anti-viral responses involving type Ι IFNs and CD4(+) T cells. We first measured cytokine production of IL-12 and type Ι IFNs from cDCs and pDCs upon stimulation with OLL1073R-1. Isolated cDCs and pDCs were stimulated with OLL1073R-1 for 24 h in separate cultures, and IL-12, IFN-α2a and IFN-β levels in supernatants were quantified. IL-12 levels in supernatants were significantly increased in both cDCs and pDCs by OLL1073R-1 stimulation (Fig 4A). In contrast, IFNα-2a and IFN-β levels in supernatants were significantly increased in pDCs, but not in cDCs (Fig 4A). These results suggested that both DC subsets contributed to CD4(+) T cell activation through IL-12 production, whereas pDCs are the primary source of type Ι IFNs in response to direct stimulation with OLL1073R-1.

**Fig 4:**
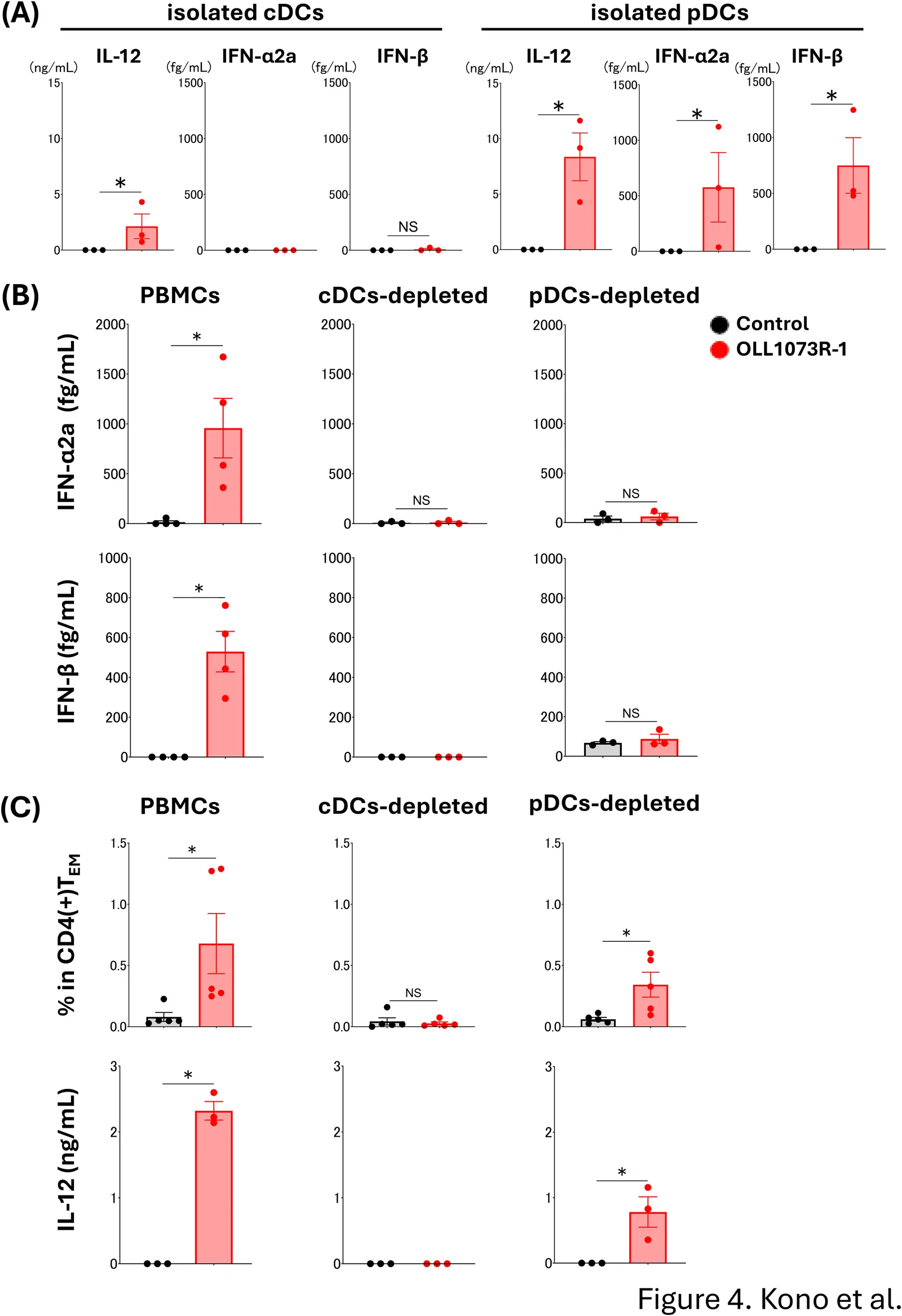
Contribution of cDCs and pDCs to type I IFN production and anti-viral CD4(+) T cell responses stimulated with OLL1073R-1. (A) Isolated cDCs and pDCs are cultured with or without *L*. *bulgaricus* OLL1073R-1 for 24 h. IL-12p40, IFN-α2a, IFN-β levels in supernatants were analyzed (n = 3). (B) PBMCs (n = 4), cDCs-depleted PBMCs (n = 3) and pDCs-depleted PBMCs (n = 3) were cultured with or without *L*. *bulgaricus* OLL1073R-1 for 24 h. IFN-α2a, IFN-β levels in supernatants were analyzed. (C) PBMCs, cDCs-depleted PBMCs and pDCs-depleted PBMCs were cultured with or without *L*. *bulgaricus* OLL1073R-1 under the stimulation of influenza antigen. The ratio of IFN-γ expressing cells in CD4(+) T_EM_ were analyzed 24 h after incubation (n = 5, *: *p* < 0.05, NS: not significant by Student’s t-test). IL-12 levels in supernatants were analyzed 20 h after incubation (n = 3). *: *p* < 0.05, NS: not significant by Mann-Whitney U test unless otherwise noted. Data below detection limit were calculated as zero.

On the other hand, isolated DC cultures do not fully reflect interaction between immune cells such as cooperative production of type Ι IFNs by cDCs and pDCs upon stimulation with lactic acid bacteria, which has been previously reported [37]. Therefore, we examined how both DC subsets contributed to anti-viral responses on stimulation with OLL1073R-1 in PBMC. To clarify the coordinate effects of cDCs and pDCs on the type I IFN response, PBMCs, cDCs-depleted PBMCs and pDCs-depleted PBMCs were stimulated with OLL1073R-1 for 24 h, and type Ι IFN production was assessed. IFN-α2a and IFN-β levels in supernatants were significantly increased in intact PBMCs but not in cDCs-depleted PBMCs and pDCs-depleted PBMCs by OLL1073R-1 stimulation (Fig 4B, C). These findings indicated that both cDCs and pDCs are necessary for type Ι IFN production in PBMCs, despite pDCs being the producer of type Ι IFNs in isolated DC culture.

We further investigated the role of these cells in anti-viral responses of CD4(+) T cells upon stimulation of OLL1073R-1. We focused on CD4(+) T_EM_ which was reported to activate during viral infections such as influenza and play a role in suppressing infections [38]. PBMCs, cDCs-depleted PBMCs and pDCs-depleted PBMCs were stimulated with OLL1073R-1 in the presence of influenza antigen. CD4(+) T_EM_ in PBMCs were analyzed by flow cytometry using gating strategies shown in S5 Fig. The ratio of IFN-γ expressing cells in CD4(+) T_EM_ was significantly increased in PBMCs and pDCs-depleted PBMCs but not in cDCs-depleted PBMCs following OLL1073R-1 stimulation (Fig 4D). Consistently, IL-12 levels in supernatants were also significantly elevated in PBMCs and pDCs-depleted PBMCs but not in cDCs-depleted PBMCs (Fig 4E). These results indicate that cDCs, but not pDCs, are essential for enhancement of anti-viral CD4(+) T cell responses induced by OLL1073R-1 through IL-12 production.

### OLL1073R-1 is phagocytosed by cDCs and pDCs

mRNA-seq analysis indicated that OLL1073R-1 activate cDCs and pDCs through recognition by TLRs. Since TLR7, 8 and 9 which are included in TLRs enriched in pDCs recognize endosomal compartments, phagocytosis of OLL1073R-1 may be required for activation of these pathways [39]. In order to assess bacterial intake, PBMCs were stimulated for 24 h by OLL1073R-1 labeled by pHrodo which changed color in low-pH phagolysosome. Flow cytometric analysis revealed that the ratio of pHrodo-positve cells was significantly increased in both cDCs and pDCs fractions (Fig 5, S6 Fig), indicating that cDCs and pDCs intake OLL1073R-1. These results suggest the possibility of endosomal TLRs in DCs to recognize OLL1073R-1.

**Fig 5:**
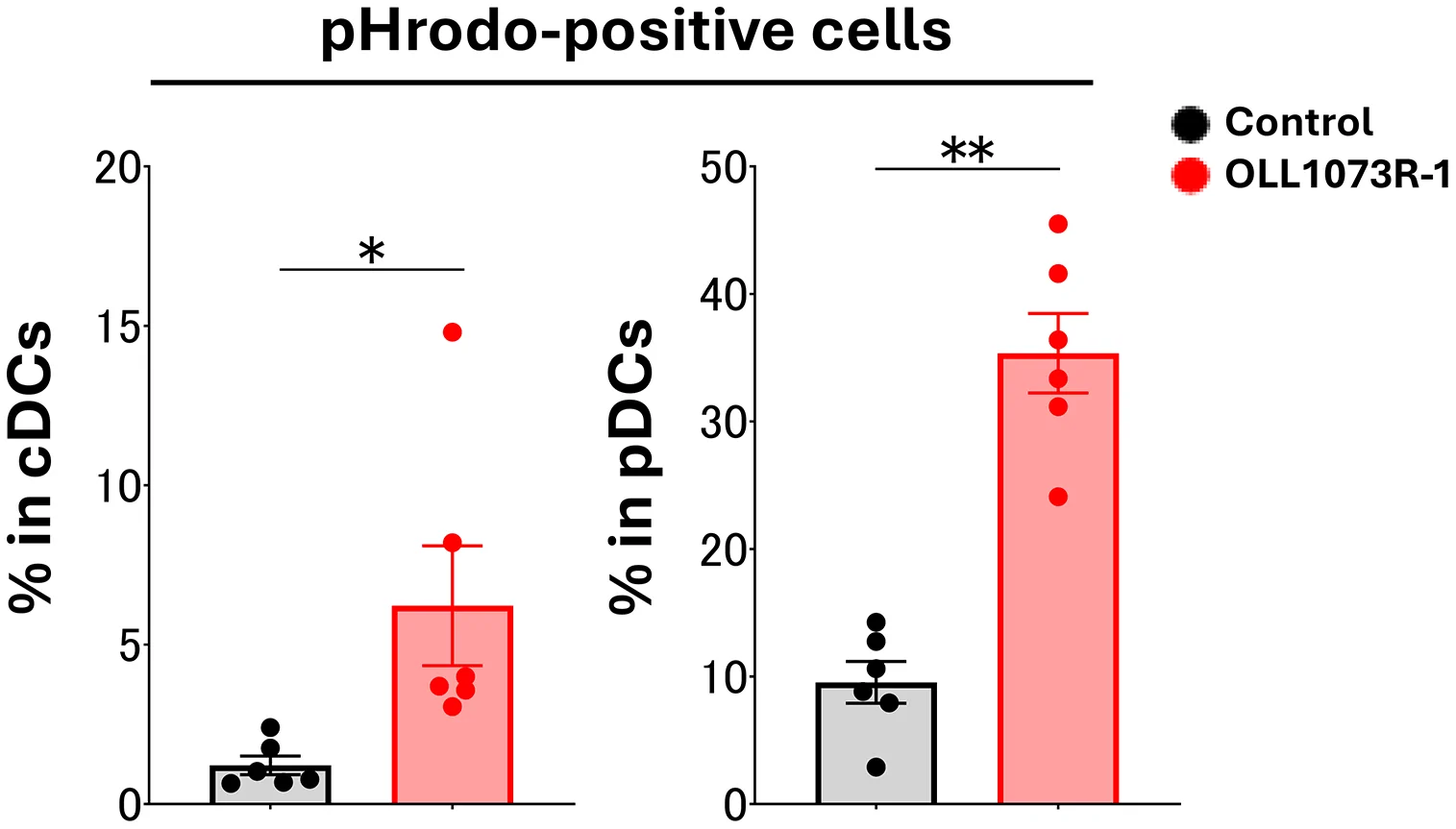
Phagocytosis of OLL1073 into cDCs and pDCs in PBMCs. PBMCs are cultured for 24 h with or without pHrodo-stained *L*. *bulgaricus* OLL1073R-1. The ratio of pHrodo-positive cells was analyzed by flow cytometry (n = 6). *: *p* < 0.05, **: *p* < 0.01 by Student’s t-test.

### OLL1073R-1 has higher capacity to activate cDCs than other *L. bulgaricus* strains

We finally compared the ability of OLL1073R-1 to activate cDCs and pDCs with that of other *L. bulgaricus* strains. PBMCs were stimulated for 24 h with OLL1073R-1, *L. bulgaricus* JCM 1002^T^ which is type strain of *L. bulgaricus* species, *L. bulgaricus* MH250033 and *L. bulgaricus* MH250034. The ratio of IL-12 expressing cDCs was significantly increased only with the stimulation of OLL1073R-1, but not other strains (Fig 6A), indicating a strain-specific capacity of OLL1073R-1 to activate cDCs. In contrast, CD86 expression in pDCs was increased upon stimulation of JCM 1002^T^ and MH250034, as well as OLL1073R-1 (Fig 6B). These results indicated that the ability of *L. bulgaricus* strains to activate pDCs is not unique to OLL1073R-1, whereas its ability to activate cDCs is strain-specific.

**Fig 6:**
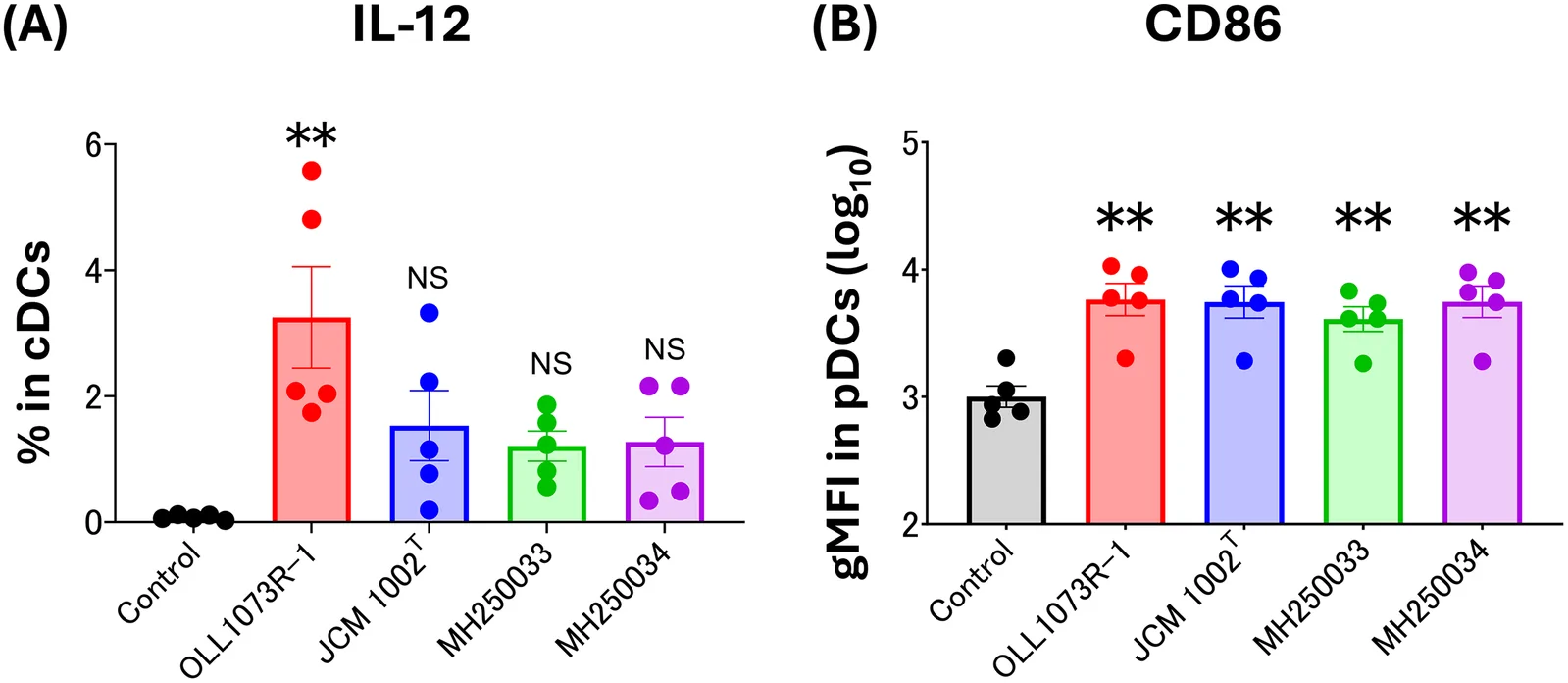
Comparison of cDCs and pDCs activation among *L. bulgaricus* strains. PBMCs are cultured with multiple *L. bulgaricus* strains for 24 h. (A) The ratio of IL-12 expressing cells in cDCs and (B) Geometric mean fluorescent intensity (gMFI) of CD86 expression were analyzed (n = 5). **: *p* < 0.01, NS: not significant by Dunnett’s test.

## Discussion

In the present study, we demonstrated that OLL1073R-1 and OLS3059 activated both cDCs and pDCs in PBMCs. Using isolated DC population, we also showed OLL1073R-1 directly act on and activate cDCs and pDCs. In addition, the experiments using cDCs or pDCs-depleted PBMCs cleared that both cDCs and pDCs were necessary for type Ι IFN production, whereas cDCs, but not pDCs, were essential for the enhancement of anti-viral responses of CD4(+) T cells with the stimulation of OLL1073R-1. These findings indicated by which mechanism lactic acid bacteria enhance anti-viral immunity through activating two different DC subsets.

Our previous report has shown that the ratio of CD86 expressing cDCs and pDCs in PBMCs was increased by the consumption of yogurt fermented with OLL1073R-1 and OLS3059 [20]. Another human study has demonstrated that intake of this yogurt increased the ratio of CD86 expressing cDCs and the change rate of CD40 expressing pDCs when stimulated by viral antigen [21]. Since our results showed the ability of OLL1073R-1 and OLS3059 to increase the ratio of CD86 and CD40 expressing cDCs and pDCs, it is suggested that both strains contained in this yogurt activate two DC subsets. In addition, both human studies have shown that DC activation was associated with the reduction of respiratory symptoms. In this study, we demonstrated the enhancement of anti-viral immune responses with the stimulation of OLL1073R-1 through activating cDCs and pDCs, which might lead to improvement of these symptoms.

Type Ι IFNs are critical mediators of anti-viral innate immune response, particularly during viral infection at early phase [36,40]. pDCs are recognized as primary sources of type Ι IFNs and are characterized by producing large amounts of type Ι IFNs [40]. In the present study, isolated pDCs, but not cDCs, produced type Ι IFNs following direct stimulation with OLL1073R-1. However, depletion experiments revealed that removal of either cDCs or pDCs from PBMC cultures impaired type I IFN production in response to OLL1073R-1. One of the reasons for this apparent contradiction is that intercellular communication between DC subsets is critical for production of type Ι IFNs in a multicellular immune environment. Previous study has shown that type Ι IFN production of pDCs stimulated with *L. lactis* strain was elevated when co-cultured with cDCs [37]. Another study has shown that *L. paragasseri* strain activated both cDCs and pDCs and induced type Ι IFN production in PBMCs [41]. Since type Ι IFNs have their character of positive feedback [42], it might be possible that type Ι IFNs produced by pDCs stimulated with lactic acid bacteria makes cDCs produce type Ι IFNs in turn. In the current study, the number of pDCs in PBMCs was much smaller than the number of isolated pDCs we used because the ratio of pDCs in PBMCs was less than 1%. Therefore, it is suggested that type Ι IFN production was not increased by individual activity of small number of pDCs but by cooperative interaction between cDCs and pDCs in PBMCs. Our results indicate that both cDCs and pDCs are required for type Ι IFN production in response to OLL1073R-1 *in vivo* environment where various immune cells are present.

Our study showed that cDCs, but not pDCs, were necessary for enhancement of CD4(+) T_EM_ activation by OLL1073R-1 when stimulated by influenza antigen. This is consistent with previous report which showed cDCs-depletion, but not pDCs-depletion, decreased IFN-γ production and body weight during influenza infection [10]. We have shown in previous study that the consumption of yogurt fermented with OLL1073R-1 and OLS3059 enhanced immune response to influenza and suppress influenza infection, which is suggested that activation of cDCs might contribute to these effects [23,24]. In contrast to cDCs, pDCs-depletion have been reported to make no significant influence on survival during other viral infections such as local infection of herpes simplex virus (HSV) −2 and lymphocytic choriomeningitis mammarenavirus (LCMV) infection, as well as influenza infection [43,44]. These findings indicate that enhancement of OLL1073R-1-mediated anti-viral immune responses through activating cDCs might be particularly crucial for suppressing some infectious diseases.

Previous reports have shown that some lactic acid bacteria activate cDCs and pDCs through several kinds of TLRs [25,26,40]. In the present study, mRNA-seq analysis revealed that TLR signaling and downstream NF-κB signaling were enriched in both cDCs and pDCs with the stimulation of OLL1073R-1. Our results further showed that both cDCs and pDCs intake OLL1073R-1; similar uptake was also observed with OLS3059 (S7 Fig). These findings suggest that not only TLRs expressing on cell surface, but also endosomal TLRs may be involved in activation of DCs by lactic acid bacteria. Indeed, it has been shown that *L. acidophilus* and *L. rhamnosus* strains activate cDCs via TLR2 which expresses on cell surface [25,26]. In the same study, phagocytosis was also required for cDCs activation [26,45]. Another study has shown that *L. lactis* strain activates pDCs via TLR9 which is an endosomal receptor [40]. However, in our experiment, there was no significant change in *TLR* 2, 4, 7 and 9 gene expressions which were included in the TLR signaling pathway identified by mRNA-seq when analyzed by qPCR (S8 Fig). The result of mRNA-seq also showed that only downstream genes of TLR signaling pathway upregulated by OLL1073R-1, such as NF-κB, AP-1, IRF7 (S9 Fig). Further researches such as detecting TLR gene expressions at earlier time point are required for identifying which kind of TLRs are related to activation of cDCs and pDCs by OLL1073R-1.

Our study showed that the ability of OLL1073R-1 to activate cDCs was higher than that of other *L. bulgaricus* strains, while OLS3059 had similar ability compared to other *S. thermophilus* strains (S10 Fig). The reason why *L. bulgaricus* strains and *S. thermophilus* strains have distinct trend for activating cDCs might be that there are differences in cell surface components such as peptidoglycan, lipoteichoic acid and exopolysaccharide (EPS) among lactic acid bacteria [46]. It has been reported that OLL1073R-1 is a *Lactobacillus* strain producing large amount of EPS with TLR agonist activity [18]. These findings suggest that OLL1073R-1 efficiently activate cDCs through enhancing TLR signaling by EPS on the surface of bacteria. In contrast, the ability of OLL1073R-1 and OLS3059 to activate pDCs was each as well as that of other *L. bulgaricus* and *S. thermophilus* strains (S10 Fig). A previous study reported that a specific *Lactococcus* strain, but not *Lactobacillus* strain was taken up by mouse bone marrow-derived pDCs and induced their activation [37]. In contrast, another study demonstrated that pDCs isolated from human PBMCs were activated by a *Lactobacillus* strain following bacteria uptake [28]. Consistent with these findings, our results also indicate that the ability of lactic acid bacteria to activate human pDCs through phagocytosis is not restricted to a single species or strain. The discrepancy among these studies may be attributable to differences in the species origin of the immune cells examined.

This study has several limitations. All experiments were conducted *in vitro*, and thus may not fully reflect the complex immune interactions occurring *in vivo*. However, our previous studies have shown that cDCs and pDCs were activated by the consumption of yogurt fermented with OLL1073R-1 and OLS3059 [20,21], which suggests that our *in vitro* results are physiologically possible. It is plausible that OLL1073R-1 and OLS3059, upon reaching the intestinal mucosa, activate resident cDCs and pDCs, thereby enhancing type I IFN production and anti-viral T cell responses in peripheral or lymphoid tissues.

## Conclusion

In conclusion, we demonstrate that *L*. *bulgaricus* OLL1073R-1 and *S. thermophilus* OLS3059 activate cDCs and pDCs. We further showed that *L*. *bulgaricus* OLL1073R-1 enhance anti-viral immune responses by activating cDCs and pDCs. Our findings provide insight into the mechanism how probiotic lactic acid bacteria enhance innate and acquired anti-viral immunity through activating DC subsets.

## Supporting information

S1 Fig Gating strategies of cDCs and pDCs fraction in PBMCs. FACS plot of representative samples were shown.
(TIF)

S2 Fig Ratio of activationmarker-expressing cDCs and pDCs in PBMCs stimulated by OLL1073R-1 or OLS3059. PBMCs are cultured with *L*. *bulgaricus* OLL1073R-1 or *S. thermophilus* OLS3059 for 24 h. The expression of CD86, CD40 in cDCs was analyzed by flow cytometry. Representative histograms (gray; isotype control; black, control; red, OLL1073R-1; blue; OLS3059) and the corresponding ratio were shown (n = 6). **: *p* < 0.01 by Dunnett’s test.
(TIF)

S3 Fig Gating strategies of sorting cDCs and pDCs from CD34(+) HPC. FACS plot of representative samples were shown.
(TIF)

S4 Fig The ratio of activation marker expressing cells of isolated cDCs and pDCs stimulated by OLL1073R-1. Isolated cDCs and pDCs are cultured with or without *L*. *bulgaricus* OLL1073R-1 for 24 h. The expression of CD86, CD40 in (A) cDCs and (B) pDCs was analyzed by flow cytometry. Representative histograms (black, control; red, OLL1073R-1) and the corresponding ratio of CD86 or CD40 expressing cells in live cells were shown (n = 6). **: *p* < 0.01 by Student’s t-test.
(TIF)

S5 Fig FACS plot of CD4(+)T_EM_ fraction in PBMCs. PBMCs, cDCs-depleted PBMCs and pDCs-depleted PBMCs were cultured with or without *L*. *bulgaricus* OLL1073R-1 in the presence of influenza antigen. FACS plot of (A) gating strategy and (B) IFN-γ expressing cells in CD4(+) T_EM_ of representative sample was shown.
(TIF)

S6 Fig FACS plot of cDCs and pDCs fraction in PBMCs which intake pHrodo-labeled OLL1073R-1. PBMCs are cultured for 24 h with or without pHrodo-stained *L*. *bulgaricus* OLL1073R-1. Representative plots in cDCs and pDCs were shown.
(TIF)

S7 Fig Phagocytosis of OLS3059 into cDCs and pDCs in PBMCs. PBMCs are cultured for 24 h with or without pHrodo-labeled *S. thermophilus* OLS3059. Representative plots in cDCs and pDCs were shown. The ratio of pHrodo-positive cells was analyzed (n = 6). *: *p* < 0.05, **: *p* < 0.01 by Student’s t-test.
(TIF)

S8 Fig TLR gene expression of isolated cDCs and pDCs stimulated by OLL1073R-1. Isolated (A) cDCs and (B) pDCs are cultured with or without *L*. *bulgaricus* OLL1073R-1 for 6 h and TLR gene expressions were analyzed by qPCR (n = 4) NS: not significant by Student’s t-test. cDNA samples were prepared from RNA samples of isolated cDCs and pDCs by using PrimeScript™ RT Master Mix (Takara Bio, Kusatsu, Japan). Gene expressions were analyzed by using TB Green® Premix Ex Taq™ II (Takara Bio) at the condition of first denature process at 95°C for 30 sec, followed by 40 cycles of denature process at 95°C for 5 sec and annealing and elongation process at 60°C for 30 sec. The expressions of *TLR2*, *TLR4*, *TLR7*, *TLR9* genes were calculated by relative standard curve method using *GAPDH* gene expression as endogenous control. Primers used for qPCR were shown in S1 Table.
(TIF)

S9 Fig TLR related pathway in enrichment analysis of RNA-seq of isolated cDCs and pDCs. “Immune response_TLR2 and TLR4 signaling” pathway ranked 16th in cDCs and “Immune response_TLR5, TLR7, TLR8 and TLR9 signaling” pathway ranked 13rd in pDCs were shown. Genes upregulated in *L*. *bulgaricus* OLL1073R-1 group were marked by red signature. Detailed legends of pathway map are available in the MetaCore reference guide. (https://portal.genego.com/legends/MetaCoreQuickReferenceGuide.pdf).
(TIF)

S10 Fig Comparison of cDCs and pDCs activation among *S. thermophilus* strains. PBMCs are cultured with multiple *S. thermophilus* strains for 24 h. (A) The ratio of IL-12 expressing cells in cDCs and (B) Geometric mean fluorescent intensity (gMFI) of CD86 expression were analyzed (n = 5). *: *p* < 0.05, **: *p* < 0.01 by Dunnett’s test.
(TIF)

S1 Table Primer sequences used in qPCR. (XLSX)
